# The effectiveness of blood glucose and blood ketone measurement in identifying significant acidosis in diabetic ketoacidosis patients

**DOI:** 10.1186/s13098-023-01176-w

**Published:** 2023-10-13

**Authors:** Eric S. Kilpatrick, Alexandra E. Butler, Sawsan Saeed, Naji Alamuddin, Stephen L. Atkin, David B. Sacks

**Affiliations:** 1 Department of Clinical Biochemistry, Sidra Medicine, Doha, Qatar; 2 Department of Postgraduate Studies and Research, Royal College of Surgeons in Ireland, PO Box 15503, Busaiteen, Adliya, Bahrain; 3 Department of Medicine, Royal College of Surgeons in Ireland, PO Box 15503, Busaiteen, Adliya, Bahrain; 4 Department of Internal Medicine, King Hamad University Hospital, Busaiteen, Adliya, Bahrain; 5 National Institutes of Health, Bethesda, MD USA

**Keywords:** Diabetic ketoacidosis, beta-hydroxybutyrate, Ketones, Bicarbonate, Acid-base status, pH

## Abstract

**Background:**

Patients with diabetic ketoacidosis (DKA), a potentially fatal complication of type 1 diabetes, have hyperglycemia, ketonemia and metabolic acidosis. Blood glucose and blood ketone results are often used to triage patients with suspected DKA. This study aimed to establish how effective blood glucose and blood ketone (beta-hydroxybutyrate, BOHB) measurements are in identifying patients with significant acidosis and sought to validate existing diagnostic BOHB thresholds.

**Methods:**

Initial Emergency Department results on 161 presumptive DKA episodes in 95 patients (42 F, 53 M, age range 14–89 years) containing a complete dataset of D (glucose), K (BOHB) and A (Bicarbonate [HCO_3_] and pH) results.

**Results:**

Blood glucose correlated poorly with BOHB (r = 0.28 p = 0.0003), pH (r= -0.25, p = 0.002) and HCO_3_ (r= -0.17, p = 0.04). BOHB, though better, was still limited in predicting pH (r = -0.44, p < 0.0001) and HCO_3_ (r = -0.49, p < 0.0001). A HCO_3_ of 18mmol/L equated to a BOHB concentration of 4.3mmol/L, whilst a HCO_3_ of 15mmol/L equated to a BOHB of 4.7mmol/L. Of the 133 of 161 events with HCO_3_ < 18mmol/L, 22 were not hyperglycemic (> 13.9mmol/L, n = 8), ketonemic (≤ 3mmol/L, n = 9) or either (n = 5).

**Conclusions:**

The commonly employed BOHB diagnostic cutoff of 3mmol/L could not be verified. Since acid-base status was poorly predicted by both glucose and BOHB, this highlights that, regardless of their results, pH and/or HCO_3_ should also be tested in any patient suspected of DKA.

## Introduction

Diabetic ketoacidosis (DKA) is a relatively common and potentially fatal complication of type 1 diabetes. Diagnosing diabetic ketoacidosis (DKA) traditionally involves the identification of hyperglycemia, hyperketonemia and metabolic acidosis, often in that order [1]. Recommended thresholds for diagnosis vary among different organizations [2]. Values most commonly used are plasma glucose > 11 or > 13.9 mmol/L, pH < 7.3, bicarbonate < 18 or 15 mmol/L and beta-hydroxybutyrate (BOHB) > 3 mmol/L [1, 3, 4].

However, with an increasing prevalence of euglycemic ketoacidosis due to factors including the use of sodium glucose transporter 2 (SGLT2) inhibitors [5], and a paucity of evidence confirming the 3mmol/L diagnostic threshold for blood ketones (BOHB) [2], a study verifying ketone thresholds and identifying the reliance that can be placed on glucose and BOHB testing in this clinical situation is warranted. The current study aimed to use real-world data from patients admitted to an Emergency Department with presumptive DKA to validate, or not, current blood ketone concentration thresholds as well as to explore how effective blood glucose and blood BOHB measurement in identifying patients with significant acidosis.

## Research design and methods

All patients who had a presumptive admission diagnosis of DKA in the Emergency Department of King Hamad University Hospital, Kingdom of Bahrain from November 2016 to January 2023 were included in this study. Only those with complete initial presentation laboratory data on admission comprising blood gas bicarbonate and pH measurement on a COBAS b221 gas analyzer platform (Roche Diagnostics, Indianapolis, IN, USA), BOHB measured on a Stat Strip Ketone meter (limits of measurement 0.1–7 mmol/L) (Nova Biomedical, Waltham, MA, USA) and blood glucose measurement using the Accu-check Inform II-Cobas (Roche Diagnostics, Indianapolis, IN, USA) were included. BOHB values recorded as ‘high” or “high > 7mmol/L” in patient records were plotted as 7 mmol/L. Serum urea and creatinine measurements were obtained when available.

Patient test results were compared to guidelines from the American Diabetes Association (ADA) [1], the Joint British Diabetes Societies (JBDS) [3], the International Society for Pediatric and Adolescent Diabetes (ISPAD) [4] and the American Association of Clinical Endocrinologists (AACE) [6].

Comparisons between analytes was by linear regression. Point of care BOHB values reported as > 7mmol/L were, for this analysis, regarded as 7mmol/L.

This retrospective study was approved by the King Hamad University Hospital ethics committee (reference 22–515).

### Statistics

Statistics (mean, SD) were calculated in Microsoft Excel (version 16.66.1; Redmond, WA, USA). Correlation analyses were performed in Graphpad Prism (version 9.4.1; San Diego, CA, USA).

## Results

Of 216 presentations, a complete dataset was found in 161 episodes among 95 adult patients (42 F, 53 M, median age 25 years, IQR 18–45 years) over the time course of the study.

Blood glucose showed limited correlation with BOHB (r = 0.28 p = 0.0003), pH (r= -0.25, p = 0.002) and HCO_3_ (r= -0.17, p = 0.04). Table 1 shows the categorization of patients into suspected, mild, moderate and severe DKA [1] according to their blood gas bicarbonate concentration, together with their corresponding BOHB and pH within these groupings.

**Table 1:** Stratification of blood gas bicarbonate analysis into mild, moderate, or severe DKA for ketones (beta-hydroxybutyrate, BOHB) and pH (all mean ± SD). *n = number of events*

	Blood gas bicarbonate (HCO_3_) (mmol/L)	BOHB (mmol/L)	pH
Suspected DKA n = 28	> 18+	3.6 ± 1.8	7.29 ± 0.05
Mild DKA n = 35	15–18	4.3 ± 2.2	7.29 ± 0.04
Moderate DKA n = 40	10-<15	5.6 ± 1.3	7.22 ± 0.06
Severe DKA n = 58	< 10	6.1 ± 1.5	7.07 ± 0.10

**Fig. 1:**
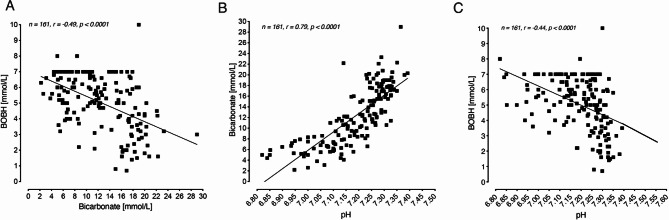
Correlation analyses of blood gas bicarbonate and ketones (beta-hydroxybutyrate, BOHB) (**A**), blood gas bicarbonate and pH (**B**) and pH and BOHB (**C**). BOHB recorded as ‘high” or “high > 7mmol/L” in patient records are plotted as 7.0 mmol/L

When bicarbonate was correlated to blood BOHB (r=-0.49, p < 0.0001), a standard bicarbonate of 18mmol/L (the ADA ‘mild DKA’ criterion [1]) corresponded to a blood BOHB concentration of 4.3mmol/L, whilst a standard bicarbonate of 15 mmol/L (the AACE, ISPAD and JBDS threshold [3, 4, 6] corresponded to a blood BOHB of 4.7mmol/L (Fig. 1A). A blood gas bicarbonate of 25.2mmol/L corresponded to a blood BOHB of 3mmol/L (Fig. 1A).

When bicarbonate was correlated to pH (r = 0.79, p < 0.0001), a blood pH of 7.35 corresponded to a standard bicarbonate of 18mmol/L, whilst a blood pH of 7.26 corresponded to a standard bicarbonate of 15mmol/L (Fig. 1B). A pH of 7.30 (the diagnostic DKA cut-off of all guidelines cited here) corresponded to a standard bicarbonate of 16 mmol/L (Fig. 1B).

When blood BOHB was correlated to pH (r=-0.44, p < 0.0001), a blood pH of 7.49 corresponded to a blood BOHB concentration of 3mmol/L (Fig. 1C), while a pH of 7.30 corresponded to a BOHB of 4.4mmol/L (Fig. 1C).

One hundred and thirty three of the 161 admission episodes suspected of DKA had bicarbonate concentrations ≤ 18mmol/L. Of these, 22 were not hyperglycemic (> 13.9mmol/L, n = 8), ketonemic (≤ 3mmol/L, n = 9) or either (n = 5). Reducing the diagnostic threshold for BOHB to ≥ 1.6mmol/L identified 130 of the 133 (98%) subjects with bicarbonate concentrations ≤ 18mmol/L but also a further 11 where bicarbonate was instead > 18mmol/L.

There was no statistically significant linear relationship between serum creatinine concentration and BOHB, pH or glucose (p = 0.45, p = 0.9, p = 0.17, respectively, n = 156), despite the potential analytical interference of ketones with serum creatinine. Similarly, there was no statistically significant linear relationship between serum urea, as a marker of circulatory volume, and BHB (p = 0.97) or pH (p = 0.86). There was a significant relationship between urea and glucose (p = 0.039) but the r squared was only 0.03.

## Discussion

This study of patients presumed as having DKA has been unable to verify the equivalence between a BOHB concentration of 3mmol/L and that of a bicarbonate concentration of 18mmol/L. Indeed, on average, pH and bicarbonate concentrations which were not abnormal equated to a BOHB of 3mmol/L. The 3mmol/L value originally adopted by several guidelines appears derived from preliminary recommendations based on a study of only 14 patients admitted to hospital with DKA [7]. A single larger study which equated BOHB concentrations to a bicarbonate concentration of 18mmol/L actually only quoted the BOHB equivalence of 3.0mmol/L to be in children, while in adults this was 3.8mmol/L [8]. Notwithstanding these data, a BOHB threshold of 3.0mmol/L continues to be promulgated in guidelines [3, 4]. In the current study, the 18mmol/L bicarbonate level equated, on average, to a BOHB concentration of 4.3mmol/L, which is closer to the aforementioned Sheikh-Ali adult value and other more contemporary findings [8, 9]. More recent DKA diagnostic guidelines have suggested a lower bicarbonate concentration of < 15mmol/L as a diagnostic criterion [3, 4, 6]. In our study, this equated to a BOHB value of 4.7mmol/L.

It might be assumed that our data therefore suggests that consideration should be given to raising the diagnostic DKA threshold for blood BOHB concentrations. However, what this current study has also shown is that in real-world situations, using modern point-of-care blood ketone testing, there is a marked spread of BOHB values around the same (including pathological) bicarbonate concentration (r=-0.49) or, alternatively, the pH (r=-0.44). This spread means that significant acidosis, for instance defined as pH < 7.30, may be present even when the BOHB concentration is considerably lower than the 4.4mmol/L equivalent value, as indicated in Fig. 1C, and, indeed, lower than the 3mmol/L traditionally used.

This study thus raises the question about what is hoped to be achieved when initially investigating possible DKA in an acute setting. Is it to establish if there is hyperglycemia present (or another cause) which is sufficient to lead to hyperketonemia and then to determine whether this has resulted in a significant metabolic acidosis (a ‘D-K-A’ strategy)? Or is it to establish the reverse, namely, if there is a metabolic acidosis present, whether ketonemia is its major (or only) source and then to determine if hyperglycemia also exists (an ‘A-K-D’ strategy)? It could be argued that any life-threatening situation is likely to be a consequence of acidosis rather than ketonemia, irrespective of whether hyperglycemia exists, although acid-base compensatory mechanisms mean pH or bicarbonate measurements alone are unlikely to be perfect triage tests either. Certainly, guidelines cannot cover every clinical presentation and, as previously pointed out [10], some, such as the ADA position statement, make clear that the criteria may be inaccurate in roughly 10% of cases [1]. In our study, 22 of 133 episodes of potential DKA (~ 17%) with a bicarbonate concentration ≤ 18mmol/L were either not diagnostically hyperglycemic (> 13.9mmol/L, n = 8) or ketonemic (≤ 3mmol/L, n = 9) or both (n = 5).

The collection of the 3 components required to meet a DKA diagnosis (namely, increased glucose, increased blood ketones and metabolic acidosis) is not consistently gathered [6] which could potentially be detrimental if it means clinical staff do not see the need to assess the acid-base status of a patient because their blood glucose and/or ketones have not themselves reached their respective diagnostic thresholds. This could delay a diagnosis or mean the patient re-presents later with more severe illness. One approach to tackle this situation is the recommendation to reduce the threshold level for acting on blood ketones, for instance by having intermediate blood ketone concentrations such as 1.6–3mmol/L [11] or even lower [6]. From our data, including patients with BOHB values of 1.6 to < 3mmol/L would have improved sensitivity by identifying 11 of the 14 significantly acidotic (bicarbonate ≤ 18mmol/L) patients ‘missed’ because they had a BOHB ≤ 3mmol/L. However, it would also have reduced specificity by including 11 other episodes where BOHB was between 1.6 and < 3mmol/L while bicarbonate was > 18mmol/L.

Euglycemic DKA, which was thought to comprise up to 10% of DKA presentations even before the advent of SGLT2 inhibitors [12], limits lowering glucose cut-offs in a similar way to BOHB. Indeed, this is the reason given for one guideline removing blood glucose altogether as a diagnostic criterion [6]. In addition, exogenous ketone bodies themselves are known to be associated with a hypoglyemic effect, so it is possible that this phenomenon could influence the association between endogenous BOHB and glucose [13].

Most patients presenting with DKA do so unequivocally, as was the case in our dataset. However, where the diagnosis is less obvious, the poor correlation between both blood glucose and blood ketones with acid-base status would suggest the only reliable means of ensuring all significantly acidotic patients are identified is by guaranteeing that pH and/or bicarbonate is measured in all patients where DKA is a possibility.

Study limitations include this not being a controlled study, as it includes all patients presenting with possible DKA; however, this can also be considered as a strength, as it presents ‘real world’ data. The same interpretation could be made of the use of a meter, rather than a laboratory instrument, to measure BOHB. In a comparison study, the Stat Strip BOHB meter showed good correlation to a central laboratory reference method and good day-to-day reproducibility [14]. Although it is not possible to estimate the fraction of blood BOHB measurements performed at point-of-care in the US, in the UK at least 76% of institutions perform point-of-care BOHB [15]. In this regard, a further strength is that the patients attended a single emergency room, so that testing was performed on the same equipment, though not necessarily by the same operator. Regarding the population studied, the majority of patients were Bahraini, though the results presented here are likely to pertain to any ethnic group, and the data included adolescent as well as adult subjects although this might still represent the typical cohort of patients presenting to many medical institutions. Lastly, being a study of just admission data, it was not possible to track the changes in BOHB, pH and glucose during treatment.

In conclusion, this study has failed to verify a 3mmol/L BOHB cut-off as being equivalent to a bicarbonate of 18mmol/L. Regardless, the range of acid-base statuses around any BOHB threshold is likely to be so large that pH or bicarbonate needs to be measured in every patient with potential ketoacidosis. With this in mind, to prioritize the relative importance of each biochemical test – and to help assure the acid-base status of a patient is always assessed - our data suggest that evaluating possible DKA should always involve assessing all three biochemical components (pH/HCO_3_, BOHB and glucose).

## Data Availability

All the data for this study will be made available upon reasonable request to the corresponding author.

## References

[1] Kitabchi AE, Umpierrez GE, Miles JM, Fisher JN (2009). Hyperglycemic crises in adult patients with diabetes. Diabetes Care.

[2] Kilpatrick ES, Butler AE, Ostlundh L, Atkin SL, Sacks DB (2022). Controversies around the measurement of blood Ketones to diagnose and manage Diabetic Ketoacidosis. Diabetes Care.

[3] Dhatariya K (2021). The management of Diabetic ketoacidosis in adults. London, UK: Joint British Diabetes Societies for Inpatient Care.

[4] Wolfsdorf JI, Glaser N, Agus M, Fritsch M, Hanas R, Rewers A (2018). ISPAD Clinical Practice Consensus Guidelines 2018: Diabetic ketoacidosis and the hyperglycemic hyperosmolar state. Pediatr Diabetes.

[5] Rosenstock J, Ferrannini E (2015). Euglycemic Diabetic Ketoacidosis: a predictable, detectable, and Preventable Safety concern with SGLT2 inhibitors. Diabetes Care.

[6] Agiostratidou G, Anhalt H, Ball D, Blonde L, Gourgari E, Harriman KN (2017). Standardizing clinically meaningful outcome measures beyond HbA(1c) for type 1 diabetes: a Consensus Report of the American Association of Clinical Endocrinologists, the American Association of diabetes educators, the american Diabetes Association, the Endocrine Society, JDRF International, the Leona M. and Harry B. Helmsley charitable trust, the Pediatric Endocrine Society, and the T1D Exchange. Diabetes Care.

[7] Wallace TM, Meston NM, Gardner SG, Matthews DR (2001). The hospital and home use of a 30-second hand-held blood ketone meter: guidelines for clinical practice. Diabet Med.

[8] Sheikh-Ali M, Karon BS, Basu A, Kudva YC, Muller LA, Xu J (2008). Can serum beta-hydroxybutyrate be used to diagnose diabetic ketoacidosis?. Diabetes Care.

[9] Hirobata T, Inaba H, Kaido Y, Kosugi D, Itoh S, Matsuoka T (2022). Serum ketone body measurement in patients with diabetic ketoacidosis. Diabetol Int.

[10] Dhatariya KK, Umpierrez GE (2017). Guidelines for management of diabetic ketoacidosis: time to revise?. Lancet Diabetes Endocrinol.

[11] Diabetes (type. 1 and type 2) in children and young people: diagnosis and management.: National Institute for Health and Care Excellence guidance 2021. Contract No.: NG18. 26334077

[12] Munro JF, Campbell IW, McCuish AC, Duncan LJ (1973). Euglycaemic diabetic ketoacidosis. Br Med J.

[13] Soto-Mota A, Norwitz NG, Evans RD, Clarke K (2022). Exogenous d-beta-hydroxybutyrate lowers blood glucose in part by decreasing the availability of L-alanine for gluconeogenesis. Endocrinol Diabetes Metab.

[14] Ceriotti F, Kaczmarek E, Guerra E, Mastrantonio F, Lucarelli F, Valgimigli F (2015). Comparative performance assessment of point-of-care testing devices for measuring glucose and ketones at the patient bedside. J Diabetes Sci Technol.

[15] Dhatariya KK, Nunney I, Higgins K, Sampson MJ, Iceton G (2016). National survey of the management of Diabetic Ketoacidosis (DKA) in the UK in 2014. Diabet Med.

